# Screening of compounds to identify novel epigenetic regulatory factors that affect innate immune memory in macrophages

**DOI:** 10.1038/s41598-022-05929-x

**Published:** 2022-02-03

**Authors:** Salisa Benjaskulluecha, Atsadang Boonmee, Thitiporn Pattarakankul, Benjawan Wongprom, Jeerameth Klomsing, Tanapat Palaga

**Affiliations:** 1 Medical Microbiology, Interdisciplinary Program, Graduate School, Chulalongkorn University, Bangkok, 10330 Thailand; 2 Center of Excellence in Immunology and Immune-Mediated Diseases, Chulalongkorn University, Bangkok, 10330 Thailand; 3 Department of Microbiology, Faculty of Science, Chulalongkorn University, Bangkok, 10330 Thailand; 4 Center of Excellence in Materials and Bio-Interfaces, Chulalongkorn University, Bangkok, 10330 Thailand

**Keywords:** Drug discovery, Immunology, Molecular medicine

## Abstract

Trained immunity and tolerance are part of the innate immune memory that allow innate immune cells to differentially respond to a second encounter with stimuli by enhancing or suppressing responses. In trained immunity, treatment of macrophages with β-glucan (BG) facilitates the production of proinflammatory cytokines upon lipopolysaccharide (LPS) stimulation. For the tolerance response, LPS stimulation leads to suppressed inflammatory responses during subsequent LPS exposure. Epigenetic reprogramming plays crucial roles in both phenomena, which are tightly associated with metabolic flux. In this study, we performed a screening of an epigenetics compound library that affects trained immunity or LPS tolerance in macrophages using TNFα as a readout. Among the 181 compounds tested, one compound showed suppressive effects, while 2 compounds showed promoting effects on BG-trained TNFα production. In contrast, various inhibitors targeting Aurora kinase, histone methyltransferase, histone demethylase, histone deacetylase and DNA methyltransferase showed inhibitory activity against LPS tolerance. Several proteins previously unknown to be involved in innate immune memory, such as MGMT, Aurora kinase, LSD1 and PRMT5, were revealed. Protein network analysis revealed that the trained immunity targets are linked via Trp53, while LPS tolerance targets form three clusters of histone-modifying enzymes, cell division and base-excision repair. In trained immunity, the histone lysine methyltransferase SETD7 was identified, and its expression was increased during BG treatment. Level of the histone lysine demethylase, LSD1, increased during LPS priming and siRNA-mediated reduction resulted in increased expression of *Il1b* in LPS tolerance. Taken together, this screening approach confirmed the importance of epigenetic modifications in innate immune memory and provided potential novel targets for intervention.

## Introduction

The innate immune response, as the first line of defense, is generally nonspecific and has been characterized as an immune response with no memory. Recent evidence, however, strongly indicates that innate immune cells, such as macrophages, neutrophils, natural killer cells or innate lymphoid cells, can exhibit characteristics of immune memory by altering the response after previous infection or vaccination^1,2^. Innate immune memory is nonspecific, maintained for a relatively short duration, and does not involve specific antigen receptors generated by gene rearrangement, as in adaptive immune memory^3,4^. Innate memory is classified into two different types, i.e., “trained immunity”, which is the heightened immune response that can induce nonspecific protection, and “tolerance”, which is the repressed immune response that manifests in cancer and immune paralysis in sepsis^1,2,5,6^. In macrophages, trained immunity can be induced by priming with fungal cell wall ß-glucan (BG), vaccination with Bacillus Calmette–Guérin (BCG), and infection with *Candida albicans*^4,6,7^. In contrast, the tolerance response can be induced by primary stimulation with potent inflammatory stimulators, most of which are pathogen-associated molecular patterns (PAMPs) or damage-associated molecular patterns (DAMPs), including ligands for Toll-like receptors (TLRs), such as lipopolysaccharide (LPS) and Pam3CysSerLys4 (Pam3CSK4), and inflammatory cytokines, such as TNFα^5,8^. Tolerance results in selective repression of a set of tolerizeable genes after restimulation with the same or different stimulants^5,9^. Trained immunity may provide protection against unrelated pathogens after vaccination, while unregulated trained immunity may result in maladaptive immune responses that aggravate chronic inflammatory conditions or autoimmune diseases. Moreover, tolerance may be pivotal for controlling a heightened immune response during sepsis, but it can have detrimental consequences during secondary infection after sepsis or in cancer patients^1,10^.

Mechanistic studies on how innate immune memory is acquired reveal that epigenetic and metabolic reprogramming play essential roles. Regulation of the expression of selective genes in trained and tolerant macrophages results from changes in chromatin structure^5,6^. The main mechanism that regulates this process is at the epigenetic level through histone modification, DNA methylation, and noncoding RNA expression^1,2,5^. Histone modification is one of the major epigenetic mechanisms that controls the induction of trained and tolerant macrophages^1,2,5,6^. This mechanism relies on histone tail marking, which has a profound impact on gene expression by changing promoter accessibility or controlling the activity of distal enhancer elements^11,12^.

Previous studies demonstrated that enhancement of the immune response in β-glucan-trained macrophages involves the deposition of active histone marks, such as acetylation of lysine 27 (H3K27ac) and monomethylation and trimethylation of lysine 4 on histone H3 (H3K4me1 and H3K4me3, respectively), in the promoters of targeted genes, including proinflammatory cytokines and intracellular signaling molecules. These modifications allow gene expression by interfering with the histone/DNA interaction, leading to a loosened chromatin structure^6,12–14^. In contrast, active histone marks in LPS-tolerant macrophages were only observed in the promoters of inflammatory genes during LPS priming, and these active histone marks were replaced with repressive epigenetic marks, such as dimethylation of lysine 9 on histone H3 (H3K9me2) and CpG methylation after restimulation with LPS^5,12,14–16^. Enzymes that are shown to be involved in epigenetic modifications during trained immunity or tolerance include histone methyltransferase (HMT), histone acetyl transferase (HAT), and DNA methyltransferase (DNMT), which are potential targets for therapeutic interventions^15–19^. Although various epigenetic regulations have been shown to be involved in innate immune memory, only limited targets for pharmacological intervention have been reported. Furthermore, the identification of additional epigenetic modifiers will be beneficial for better understanding how innate immune memory is regulated.

In this study, we performed a screening of an epigenetics compound library to identify additional novel epigenetic modifications that control trained immunity and tolerance in macrophages using BG-trained or LPS-tolerant models. We have confirmed the known epigenetic modifying enzymes that have been previously shown to regulate trained immunity and tolerance in this study. More importantly, we have identified previously unknown potential novel compounds that have not been previously documented to be involved in innate immune memory. Detailed studies into the mode of action of these compounds may alter innate immune memory in macrophages and provide novel intervention strategies.

## Materials and methods

### Animals

Eight-week-old female C57BL/6 mice were used in this study (Nomura Siam International, Thailand). All experimental procedures involving laboratory animals were approved by the Institutional Animal Care and Use Committee (IACUC) of the Faculty of Medicine, Chulalongkorn University (approval protocol No. 025/2562). All experiments were performed according to the guidelines issued by the IACUC.

### Generation of bone marrow-derived macrophages (BMM)

BMMs were generated from bone marrow cells extracted from the tibia and femur of C57BL/6 mice. Bone marrow cells were cultured in Dulbecco's modified Eagle’s medium (DMEM) (HyClone, USA) supplemented with 10% (v/v) fetal bovine serum (Gibco, USA), 10 mM HEPES (HyClone, USA), 1 mM sodium pyruvate (HyClone, USA), 100 U/ml penicillin, and 0.25 mg/ml streptomycin (DMEM complete media) with 20% L929 culture supernatant and 5% horse serum (HyClone, USA), and fresh media was added at day 4. After 7 days in culture, cells were detached with cold PBS and stored at − 80 °C until use. BMMs were confirmed by flow cytometry using the macrophage cell surface markers F4/80 and CD11b^20^.

### Induction of beta-glucan (BG)-trained and LPS-tolerant macrophages

For the induction of BG-trained or LPS-tolerant macrophages, BMMs were cultured in complete DMEM and primed with 50 µg/ml pachyman BG (Megazyme, USA) for trained macrophages or 100 ng/ml *Escherichia coli* LPS (L2880, Sigma Aldrich, USA) for tolerant macrophages. After 24 h of priming, the medium was replaced with fresh DMEM complete medium and the cells were rested for 48 h. The resting step was followed by LPS (10 ng/ml) stimulation for the indicated times. Culture supernatant, RNA or cell lysates were harvested at the indicated times for analysis. The amount of TNFα in the culture supernatant was measured by a mouse TNFα ELISA kit (Biolegend, USA) according to the manufacturer’s protocol.

### Epigenetics compound library screening

The Epigenetics Compound Library with a unique collection of 181 epigenetics compounds (Cat L1900, Selleckchem, USA) was used as the inhibitor source. The library was purchased and obtained in January 2018 and the list of compounds in the library was shown in Supplementary Table 1. For the screening assay, BG-trained or LPS-tolerant macrophages were pretreated with the inhibitors at two concentrations of inhibitory concentration 50 (IC50) from the manufacturer’s data for 1 h and during the priming or stimulation phase. Control cells received vehicle control DMSO and were subjected to the same priming. Cells were cultured in the presence of inhibitors during priming or stimulation for 24 h. The culture supernatant was subjected to ELISA to measure TNFα. The relative amount of TNFα was calculated as the fold change of the inhibitor-treated cells compared with the vehicle control-treated cells. The inhibitors that showed enhancing or suppressing effects with fold changes of 1.5-fold or higher and 0.75-fold or lower, respectively, were chosen for further confirmation. Interaction of the potential targets identified in this study was performed with STRING version 11.0 (https://string-db.org/)^21^.

### Western blotting

BMMs were treated to become BG-trained or LPS-tolerant macrophages as described above. Cell lysates were collected at the indicated times using RIPA buffer (50 mM Tris HCl pH 7.4, 150 mM (for other proteins) or 500 mM (for histone, mTOR and LSD1 extraction) NaCl, 5 mM EDTA, 1% nonidet P-40, 0.5% sodium deoxycholate supplemented with protease and phosphate inhibitors (Cell Signaling Technology, USA)). The protein concentrations were measured by a bicinchoninic acid assay using the Pierce BCA Protein Assay Kit (Thermo-Fisher Scientific, USA). Proteins were resolved by 6% (mTOR), 8% (LSD1), 10% (Aurora kinase or SETD7) or 15% (histone) SDS-PAGE and subjected to Western blot as described elsewhere. The antibodies were diluted in PBS with 3% (w/v) skim milk at the following concentrations: rabbit anti-H3K4me3 antibody, 1:1000; rabbit anti-H3K27me3 antibody, 1:1000; rabbit anti-total H3 antibody, 1:4000; rabbit anti-phospho mTOR, 1,1000; rabbit anti-mTOR, 1,1000; rabbit anti-phospho Aurora A/Aurora B/Aurora C, 1:1,000, rabbit anti-LSD1, 1:2000 and goat anti-rabbit IgG HRP, 1:4000 (all antibodies were from Cell Signaling Technology, USA); mouse anti-actin antibody, 1:10,000 (Merck Millipore, USA), rabbit anti-GAPDH antibody, 1:4000 and mouse anti-SETD7 antibody, 1:2000 (Bio-Rad, USA); and sheep anti-mouse IgG HRP, 1:4000 (GE Healthcare Life Sciences, USA). The signal was detected by the ECL chemiluminescent detection method. Relative intensity was analyzed by ImageJ analysis.

### Quantitative reverse transcription realtime-PCR (qRT-PCR)

Total RNA of macrophages treated as indicated was harvested with the TRIzol reagent (Invitrogen, USA) and extracted with direct-zol RNA kits (Zymo Research, USA). The quality and concentrations of RNA were measured by a NanoDrop™ 2000 spectrophotometer (Thermo Fisher Scientific). One hundred nanograms of RNA per sample was converted to cDNA, which was used for quantitative PCR using iQ™ SYBR Green SuperMix (Bio-Rad, USA) according to the manufacturer’s instructions. The primers used in this study are shown in Supplementary Table 2. The relative expression of all target genes was normalized to the expression of *Actb* by the 2^−∆∆CT^ method.

### MTT assay

BG-trained or LPS-tolerant macrophages were treated with inhibitors during priming or stimulation as indicated. After 20 h of inhibitor treatment, MTT reagent was added to a final concentration of 0.5 mg/ml and the cells were further incubated for 4 h. After incubation, 200 µl of DMSO was added to each well to dissolve the MTT formazan pellet. The intensity of the pellet was measured by a microplate reader at a wavelength of 540 nm.

### BrdU cell proliferation assay

Cell proliferation was detected by BrdU cell proliferation assay (Sigma Aldrich, USA) according to the manufacturer’s protocol. In brief, BrdU solution was added to unstimulated or LPS-tolerant macrophages 24 h before detection as indicated. After incubation and fixation, a BrdU detection antibody and IgG peroxidase-conjugated secondary antibody were added. The level of BrdU incorporation was measured by a colorimetric method at a wavelength of 450 nm using a microplate reader.

### siRNA mediated gene silencing of *lsd1*

ON-TARGET*plus*™ SMARTpool siRNA targeted murine *lsd1* or control non-targeting siRNA (NT) were purchased from Dharmacon™ (Horizon Discovery, UK). Lipofectamine 2000 (Promega, Wisconsin, USA) were used as transfection reagent. Lipid-siRNA complex was prepared in warmed Opti-MEM™ I Reducing-Serum Media (Gibco, USA) and incubate for 15 min with gently rotation before topping up to BMMs in antibiotic free DMEM complete media. The final concentration of siRNA and Lipofectamine are 50 nM and 0.6%. Following the incubation for 6 h, transfection media were replaced with fresh BMM media with antibiotic. The reduction of LSD1 mRNA and protein was confirmed at 48 h after siRNA transfection by qRT-PCR and Western blot as described above.

### Chromatin immunoprecipitation (ChIP) assay

Approximately 7.0 × 10^6^ cells of BMMs were prepared and activated as indicated. The SimpleChIP^®^ Enzymatic Chromatin IP Kit (Cell Signaling Technology, USA) was used to perform ChIP according to the manufacturer's instructions. Samples were subjected to immunoprecipitation using either Rabbit anti-H3K4me3 antibody or a control IgG antibody (Cell Signaling Technology). Fragmented DNAs were purified using spin columns (Cell Signaling Technology) and was used as the templates for qPCR using indicated primer sets spanning the *tnf-*α and *il6* promoters (Supplementary Table 2). Fold enrichments were normalized and calculated based on the total amount of 10% input presented in relative quantification using 2^−∆∆ct^ method.

### Statistical analysis

All experiments were performed in triplicate and at least twice independently, except for the primary screening. Statistical analyses were performed using GraphPad Prism version 9.0. One-way ANOVA with Dunnett’s or Tukey’s multiple comparison test and two-tailed unpaired t-test (α = 0.05) were used when comparing the two conditions. This study is reported in accordance with ARRIVE guidelines.

## Results

### BG-trained or LPS-tolerant macrophages and global changes in histone marks

**Figure 1:**
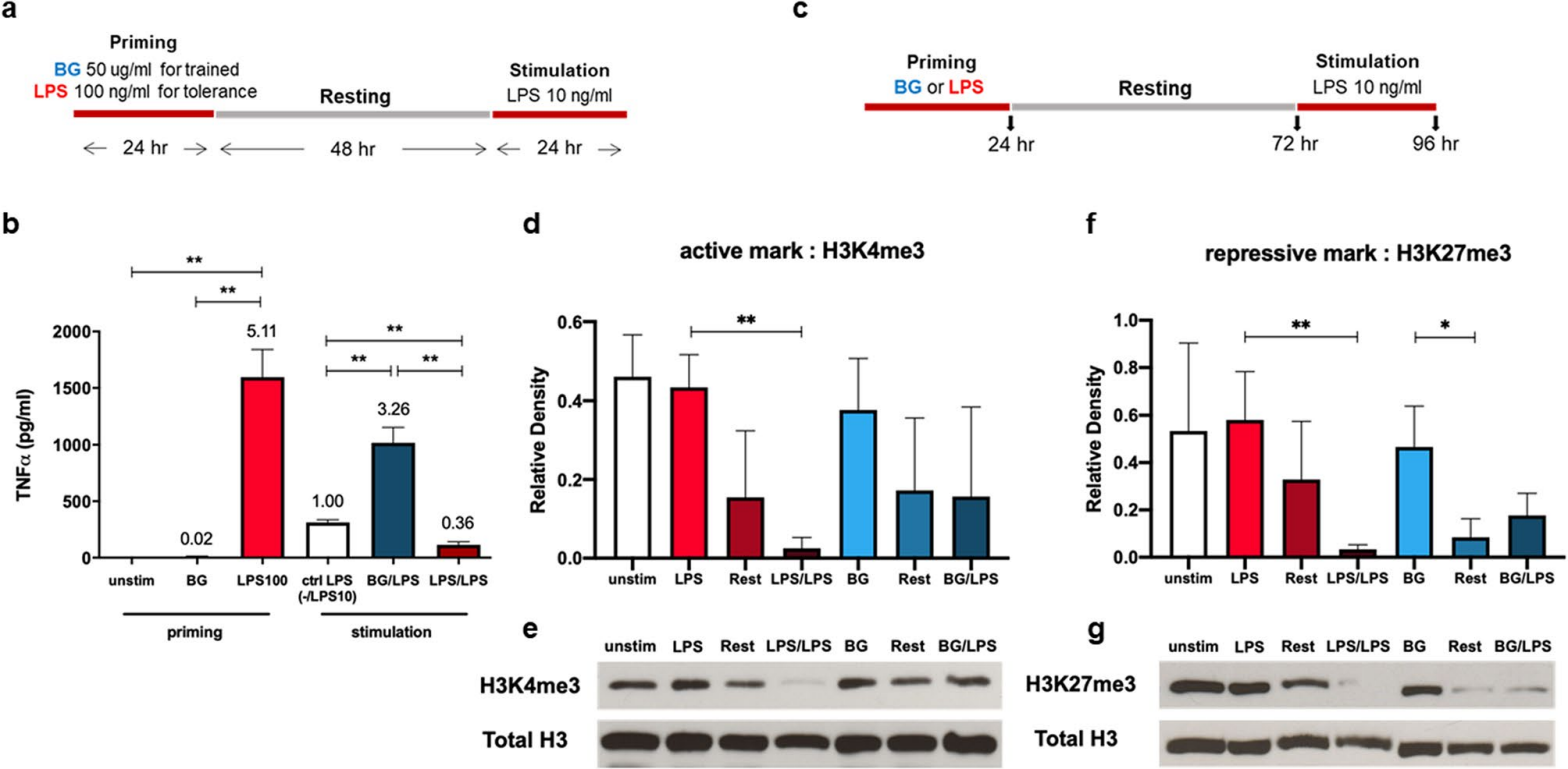
Generating BG-trained and LPS-tolerant macrophages. (**a**) Protocol to induce BG-trained or LPS-tolerant macrophages using BMM. (**b**) TNFα production was detected by ELISA in unstimulated cells, BG-primed cells, or LPS-primed cells at 24 h and in LPS-stimulated naïve cells (crtl), LPS-stimulated BG-primed cells (trained) or LPS-stimulated LPS-primed cells (tolerant) after 24 h of stimulation. The relative fold changes were calculated by normalizing to the amount obtained from LPS-stimulated naïve BMMs (10 ng/ml). (**c**) Protocol to induce BG-trained or LPS-tolerant macrophages using BMM and the indicated time for cell lysate collection. (**d**–**g**) Levels of H3K4me3 and H3K27me3 normalized to total histones in BMMs treated as indicated in (**c**) as detected by Western blot. *, **, and *** indicate significant differences compared by two-tailed unpaired t-tests at p < 0.05, p < 0.01 and p < 0.001, respectively.

To generate BG-trained or LPS-tolerant macrophages, BMMs were primed with BG or LPS for 24 h and allowed to rest in media for 48 h, as indicated in Fig. 1a. The resting step was followed by LPS stimulation (10 ng/ml) for 24 h. The amount of TNFα in the culture supernatant was measured by ELISA, and the relative levels were calculated by normalization to the amount of TNFα produced by naïve BMMs without priming that received LPS stimulation (10 ng/ml). As shown in Fig. 1b, BG priming alone minimally induced TNFα production, whereas priming with LPS at 100 ng/ml induced significantly higher TNFα than BG priming. Stimulation of BG-primed macrophages with LPS resulted in 3.26-fold higher TNFα than that in LPS-stimulated macrophages. BG-trained macrophages also enhanced the RNA expression of the proinflammatory cytokines *Tnf*, *Il6*, and *Il1b,* while no effect was observed on the level of the anti-inflammatory cytokine *Il10* (Supplementary Fig. 1a–d). Furthermore, activation of the mTOR pathway was clearly detected in BG-trained macrophages, consistent with previous studies indicating the role of the mTOR pathway in the regulation of trained immunity (Supplementary Fig. 2a,b)^22^.

For LPS-tolerant macrophages, LPS-primed BMMs produced significantly lower TNFα upon LPS stimulation than naïve BMMs receiving LPS stimulation at 10 ng/ml (0.36-fold, Fig. 1b). This reduction was also observed at the mRNA level of both proinflammatory and anti-inflammatory cytokine genes (Supplementary Fig. 1a–d). LPS priming also repressed the expression of other genes that are characterized as tolerizeable genes (T-gene), such as *Cd40* and *Serpine1* (Supplementary Fig. 2c,d). This result strongly confirmed that repeated stimulation with LPS resulted in LPS-tolerant macrophages. In contrast, the expression of non-tolerizeable genes (NT genes), such as cathelicidin antimicrobial peptide (*Camp*) and macrophage receptor with collagenous structure (*Marco*), was not only repressed but also enhanced in LPS-tolerant macrophages (Supplementary Fig. 2e,f). These results are consistent with the specific pattern of gene regulation in LPS-tolerant macrophages in a previous study^5^.

As histone modifications are one of the key mechanisms for regulating innate immune memory, we investigated the global changes in some key histone marks during BG-trained or LPS-tolerant treatment. BMMs were treated as indicated in Fig. 1c, and the total cell lysates were analyzed for representative active and repressive histone marks, H3K4me3 and H3K27me3. Priming with LPS or BG did not significantly alter the levels of H3K4me3 and H3K27me3 compared to the unstimulated condition. During the resting period or LPS stimulation in LPS-tolerant macrophages, these marks completely disappeared in LPS-tolerant cells. In contrast, these marks were still detectable but with lower intensity in BG-trained macrophages (Fig. 1d–g and Supplementary Fig. 6). Taken together, BMM-derived macrophages were successfully conditioned to become BG-trained and LPS-tolerant macrophages, and drastic changes in global representative histone marks during induction were evident.

### Screening of epigenetics compound library

To identify epigenetic modifier(s) that target molecules with a role in regulating innate immune memory in macrophages, screening assays were performed using an epigenetics compound library in BG-trained or LPS-tolerant macrophages as described above. The detailed categories of the compounds in the library are listed in Supplementary Fig. 3 and Supplementary Table 1. Among these compounds, the targets of action included histone modifying enzymes (38%), epigenetic reader domains (9%), DNA methyltransferases (4%), the JAK/STAT pathway (12%) and other kinases (13%). The screening protocol is summarized in Supplementary Fig. 4a,b. The screening aimed to identify compounds that act at the priming or stimulating step during BG-trained or LPS-tolerant induction.

For treatment at the priming step, cells were pretreated with the compounds or vehicle control DMSO for 1 h before priming with BG (50 μg/ml; trained) or LPS (100 ng/ml; tolerance) for 24 h. After media containing BG or LPS together with the compounds were removed, fresh media were added, and the cells were allowed to rest for 48 h. After the resting period, the cells were stimulated with LPS (10 ng/ml) for 24 h. For treatment during the stimulating step, cells were primed and rested as described above in the absence of compounds. One hour before LPS stimulation, BG-primed or LPS-primed BMMs were pretreated with the compounds, and LPS stimulation was carried out as described above in the presence of compounds. The readout for the screening assay was the amount of TNFα compared to the BG-trained or LPS-tolerant macrophages treated with vehicle control.

**Figure 2:**
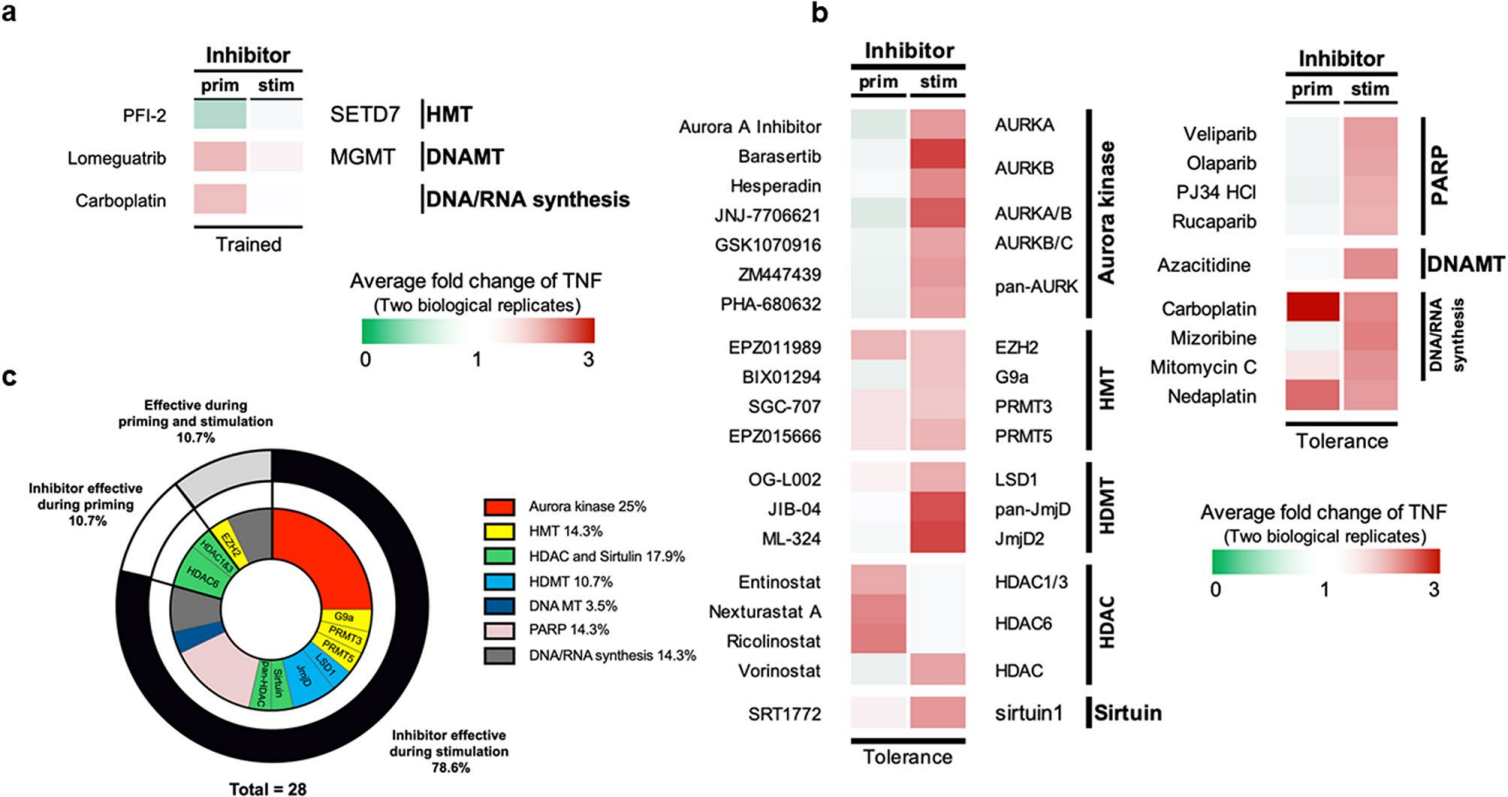
Compounds that showed enhanced or suppressed TNFα production in BG-trained and LPS-tolerant macrophages. (**a**) The relative fold changes of TNFα produced from compound-treated BG-trained macrophages (priming step or stimulation step) were calculated by normalizing to the amount of TNFα produced from vehicle control treatment of BG-trained macrophages. Average fold changes of two biological replicates are shown as heatmap format. Only compounds that increased the relative fold changes of TNFα more than 1.5-fold or lower than 0.75-fold are shown. (**b**) The relative fold changes of TNFα produced from compound-treated LPS-tolerant macrophages (priming step or stimulation step) were calculated by normalizing to the amount of TNFα produced from vehicle control treatment of LPS-tolerant macrophages. The fold changes are the average of two biological replicates and shown as heatmap format. Only compounds that increased the relative fold changes of TNFα more than 1.5-fold are shown. (**c**) Targets of suppressing inhibitors were classified based on function and time point.

The primary screening was performed using two times the IC50 concentration from the manufacturer’s information for each compound. The secondary screening was performed on those compounds that met the criteria set for the primary screening. Among the 181 compounds tested, two compounds showed enhancing effects, while only one compound showed an inhibitory effect on the BG-trained responses. PFI-2 HCl, a histone methyltransferase inhibitor, reduced trained TNFα production when applied during priming. An O^6^-methylguanine-DNA methyltransferase (MGMT) inhibitor and a DNA/RNA synthesis inhibitor enhanced TNFα production when applied during the priming step. However, none of the compounds had an effect when used during the stimulation phase (Fig. 2a and Supplementary Table 3).

In contrast, 28 compounds showed suppressive effects against LPS tolerance, which resulted in increased TNFα production after LPS stimulation. A clear inhibitory effect was observed with inhibitors targeting Aurora kinases, histone methyltransferases (HMT), histone demethylase (HDMT), histone deacetylases (HDAC), sirtuin, poly (ADP-ribose) polymerase (PARP), DNA methyltransferase and DNA/RNA synthesis. Most inhibitors rescued TNFα production under LPS tolerance conditions when added during the LPS stimulation phase (78.6%). The HDAC6 inhibitors ricolinostat and nexturastat and the HDAC1 and HDAC3 inhibitors entinostat showed inhibitory effects when added during the priming step. However, the effect of the enhancer of zeste homolog 2 (EZH2) inhibitor EPZ011989 and the DNA/RNA synthesis inhibitors carboplatin and nedaplatin were detected when treated at either the priming or the stimulating step (Fig. 2b,c). The compounds that showed enhancing effects against LPS tolerance were not further validated because the levels of TNFα were already extremely low in LPS-tolerant macrophages.

### Interacting networks of the proteins targeted by the compounds identified in the screening

**Figure 3:**
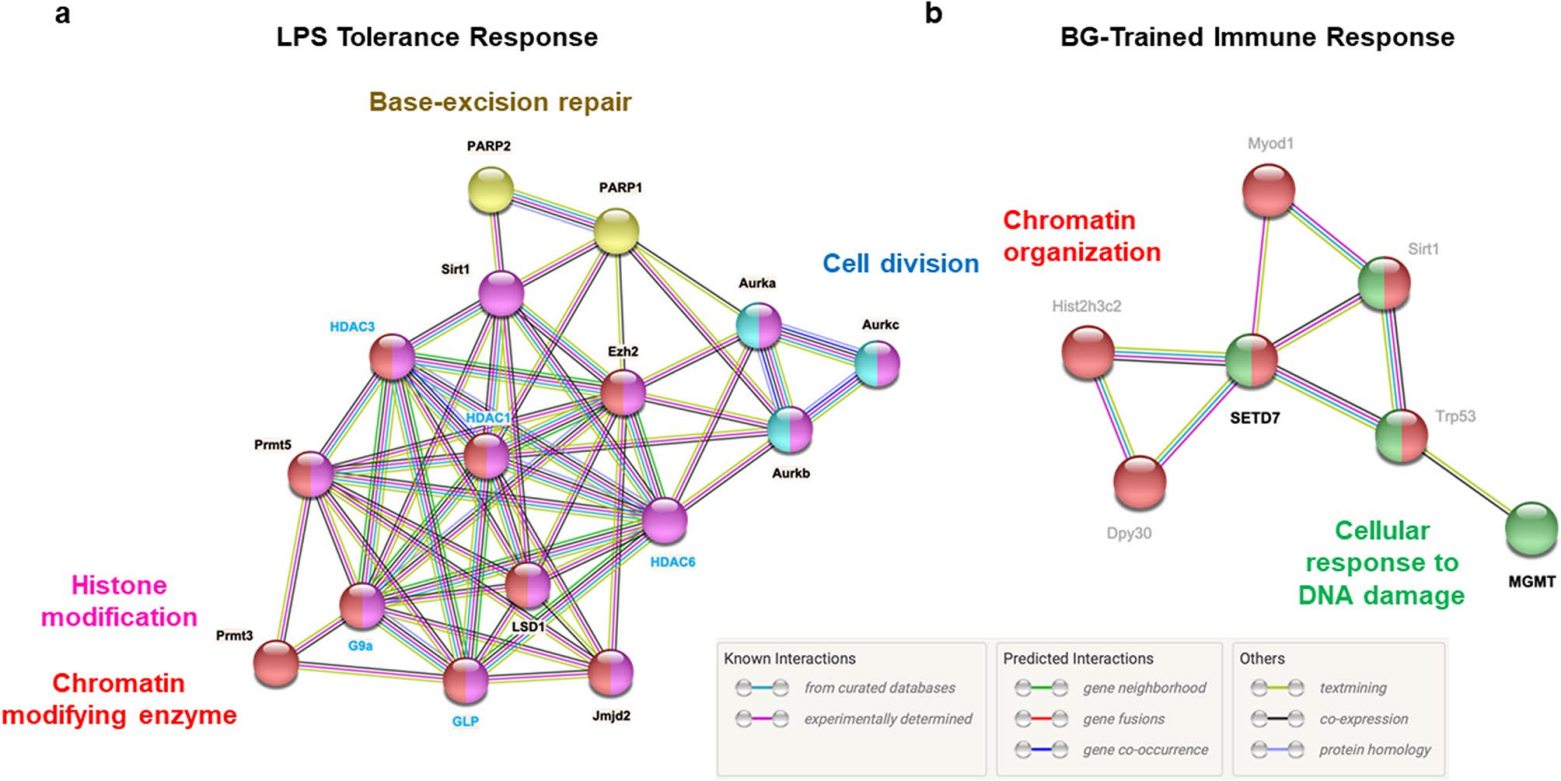
Possible interactions among the target proteins for the identified inhibitors. Potential interactions among target proteins of inhibitors identified in the screening for tolerance (**a**) and trained immunity (**b**) by STRING. The target proteins of inhibitors identified in this study are shown in black text while the potential functional partner proteins are shown in gray text. The interactions among proteins that are experimentally determined are linked with pink lines while the interactions predicted from curated database are shown in blue. Effective targets related to previous studies are represented in blue text.

To investigate the potential interaction (direct interaction or functional interaction) among the protein targets of the compounds identified by this screening, the STRING database was used to generate a protein–protein interaction network. As shown in Fig. 3a, the interaction network generated from the targets identified in LPS-tolerant macrophages revealed the following three distinctive clusters: the Aurora kinase (cell division)-related interacting network, the histone modifying enzyme network and the base-excision repair network. In contrast, for the target proteins of compounds identified in the BG-trained macrophages, the network revealed a link with Trp53, Foxo3, Suz12, Hist2h3c2 and Msh16, which share common features related to apoptosis, DNA repair and polycomb repressive complex (PRC) 2 (Fig. 3b). These analyses suggest that proteins involved in apoptosis, DNA repair, cell division and histone modification may play roles in innate immune memory in macrophages.

**Figure 4:**
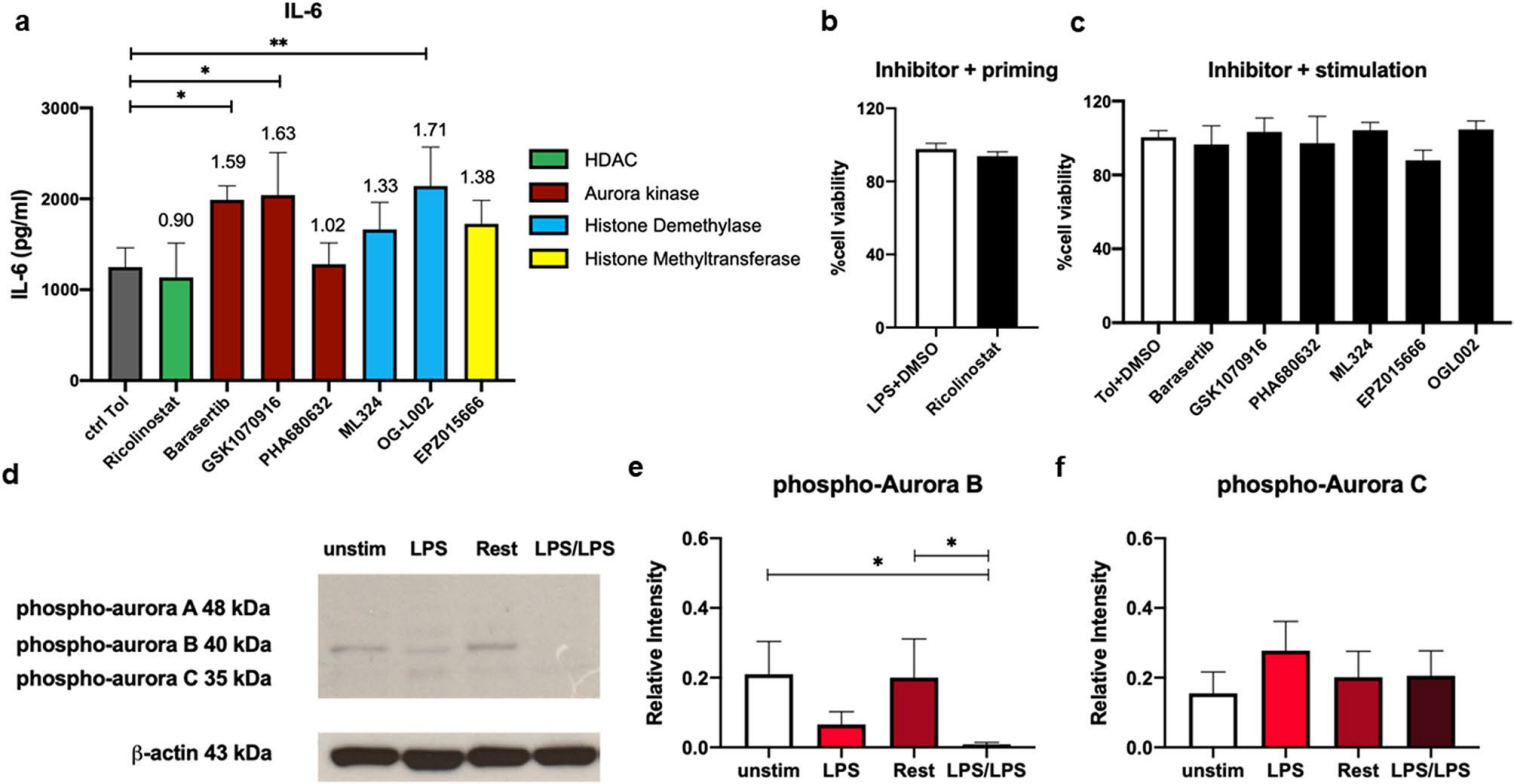
Expression profiles of Aurora kinases in LPS-tolerant macrophages. LPS-tolerant macrophages were prepared as described above. (**a**) IL-6 production after treatment with selected inhibitors from Fig. 3a was measured by ELISA at 24 h after LPS stimulation. (**b, c**) Cell viability was detected by MTT assay at 24 h after treatment with inhibitors from (**a**). (**d**-**f**) Phosphorylation of Aurora kinases was analyzed by Western blot. The relative intensity from Western blot was quantitated by ImageJ analysis and normalized to β-actin. *, **, *** and **** indicate significant differences compared by one-way ANOVA with Dunnett’s multiple comparison test (**a**) and two-tailed unpaired t-test (**d**) at *p* < 0.05, *p* < 0.01, *p* < 0.001 and *p* < 0.0001, respectively.

To confirm the effect of suppressive compounds against LPS tolerance, we investigated the level of IL-6 production after treatment with selected inhibitors, as shown in Fig. 4a. The Aurora-B-specific inhibitor barasertib and the Aurora-B/C inhibitor GSK1070916 significantly increased IL-6 production during LPS stimulation without reducing cell viability to less than 80% (Fig. 4b,c). Because the majority of the compounds that showed suppressive effects against LPS tolerance were Aurora kinase inhibitors (25%, Fig. 2b,c) and these inhibitors suppressed tolerance when using TNFα and IL-6 as readouts, we investigated the expression profiles of Aurora kinase A, B and C during priming, resting and stimulation in LPS-tolerant macrophages by Western blot. The protein bands corresponding to phosphorylated Aurora-B and Aurora-C were clearly observed, while phosphorylated Aurora-A was undetectable (Fig. 4d and Supplementary Fig. 7). The level of phosphorylated Aurora-B was significantly decreased during LPS priming and LPS stimulation, while unstimulated cells and cells in the resting period maintained Aurora-B at high levels (Fig. 4d,e). Phosphorylated Aurora-C was slightly increased during LPS priming (Fig. 4d,f). As Aurora kinases play an important role in chromatid segregation during cell division, we performed a cell proliferation assay using bromodeoxyuridine (BrdU) during DNA synthesis. The level of BrdU was significantly decreased in a time-dependent manner during LPS tolerance, indicating that cell proliferation is suppressed during LPS tolerance (Supplementary Fig. 5). These results emphasized the role of Aurora kinases in the regulation of LPS tolerance, which may not be related to cell cycle progression.

### Validating the roles of histone methyltransferase SETD7 and histone lysine demethylase LSD1 in BG-trained and LPS tolerance

**Figure 5:**
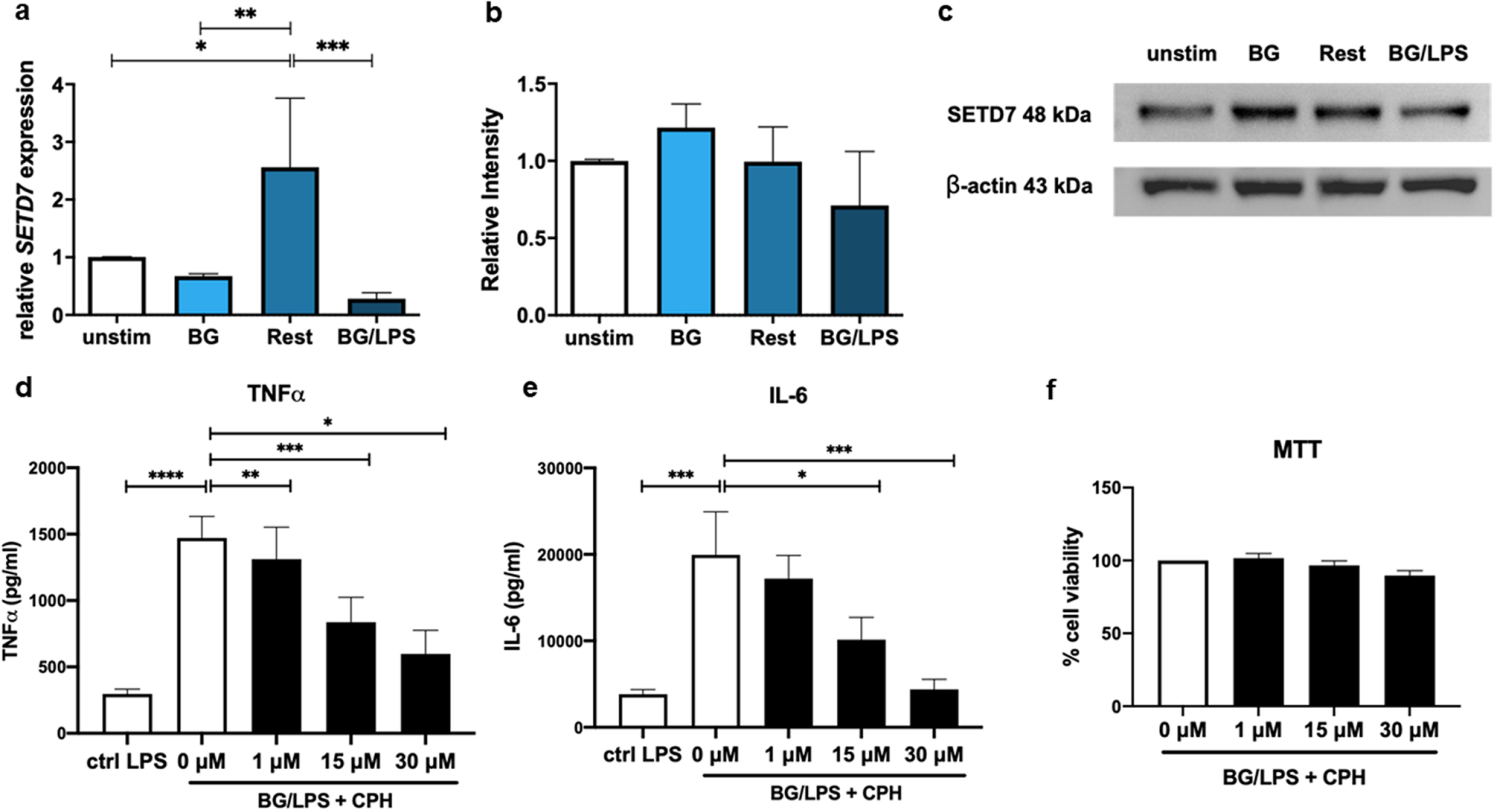
Expression profiles of SETD7 during BG-trained responses in macrophages. BG-trained macrophages were prepared as described above. (**a**) The mRNA expression profile of *Setd7* was detected by qRT-PCR. (**b**, **c**) Cell lysates were analyzed by Western blot. The relative intensity of SETD7 from Western blot was quantitated by ImageJ analysis and normalized to β-actin. The relative intensity was calculated using unstimulated samples as the baseline. (**d**, **e**) Effect of cyproheptadine (CPH) on TNFα and IL-6 production in BG-trained macrophages after LPS stimulation. (**f**) Cell viability from the MTT assay in BG-primed macrophages after treatment with different concentrations of CPH for 24 h. *, **, *** and **** indicate significant differences compared by one-way ANOVA with Tukey’s multiple test at *p* < 0.05, *p* < 0.01, *p* < 0.001 and *p* < 0.0001, respectively.

Among the targets identified in the BG-trained response, inhibition of histone lysine methyltransferase SETD7 by PFI-2 HCl showed a suppressive effect. SETD7 has many target substrates, including histones, and has only recently been implicated in regulating trained immunity^23^. We further examined the expression of *Setd7* mRNA and SETD7 protein in BG-trained macrophages. As shown in Fig. 5a, *Setd7* mRNA increased significantly at 72 h after BG priming during the rest period, whereas LPS stimulation significantly decreased its level. In contrast, the protein level of SETD7 clearly increased during BG priming and was slightly reduced but remained high during resting and LPS stimulation (Fig. 5b,c and Supplementary Fig. 7). The inhibitory effect of SETD7 on BG-trained response was confirmed by cyproheptadine (CPH), another SETD7 inhibitor. Treatment with CPH decreased BG-trained immune response as indicated by reduction in both TNFα and IL-6 production in a dose-dependent manner with little impact on cell viability (Fig. 5d–f). This result agreed with its possible role during BG priming to condition macrophages for trained responses^18^.

**Figure 6:**
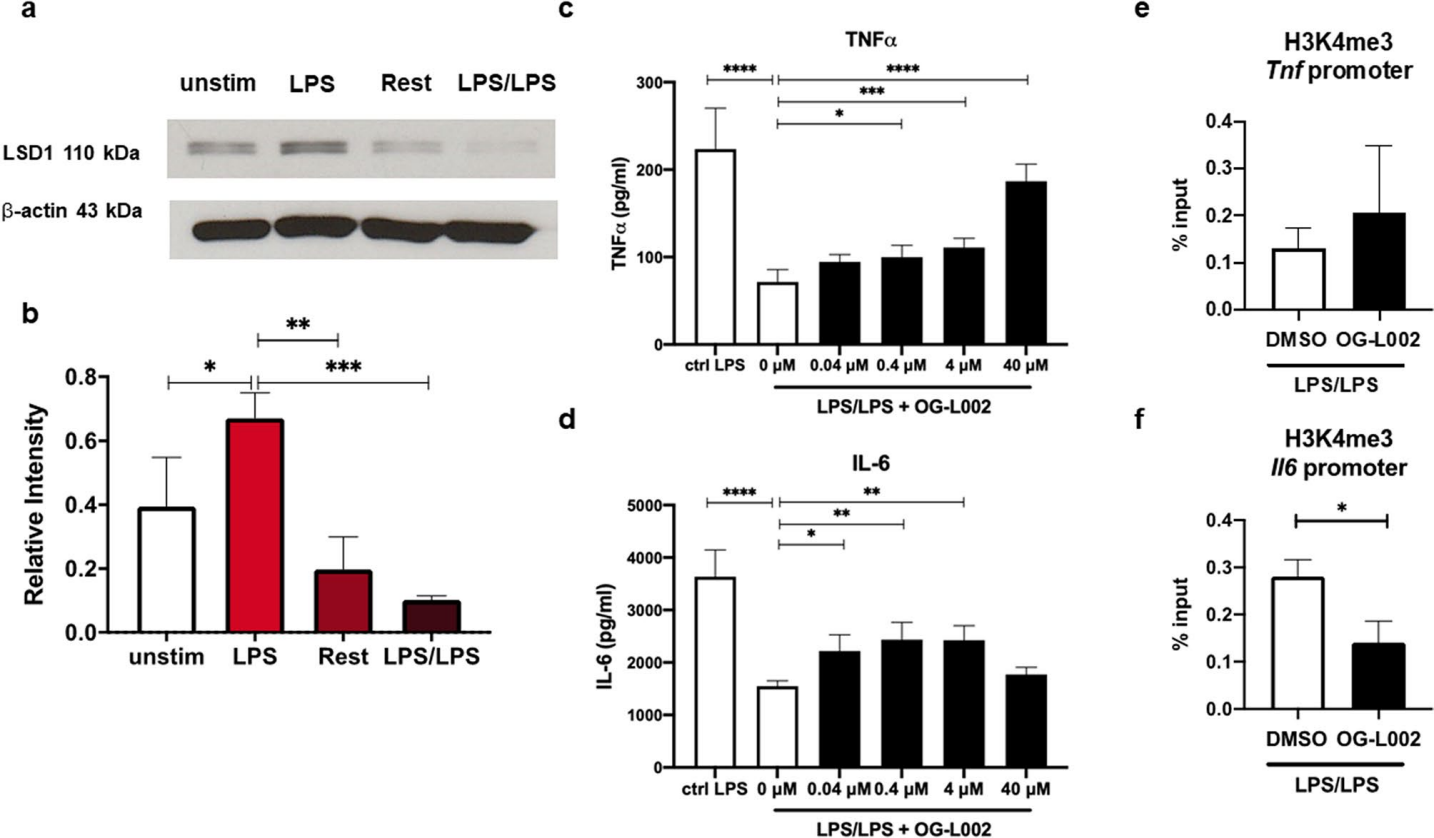
Expression profiles of LSD1 during LPS tolerance in macrophages. BMMs were induced to become LPS tolerance as described above. (**a**, **b**) Expression profiles was analyzed by Western blot. The relative intensity of LSD1 from Western blot was quantitated by ImageJ analysis and normalized to β-actin. (**c**, **d**) Effect of OG-L002 on TNFα and IL-6 production in LPS-tolerant macrophages. (**e**, **f**) Effect of OG-L002 on H3K4me3 enrichment in *Tnf* and *Il6* promoter of LPS-tolerant macrophages at 6 h after LPS stimulation. *, **, *** and **** indicate significant differences compared by one-way ANOVA with Tukey’s multiple test (**c,d**) and two-tailed unpaired t-test (**e**, **f**) at *p* < 0.05, *p* < 0.01, *p* < 0.001 and *p* < 0.0001, respectively.

Next, we focused on LSD1, a lysine demethylase of which inhibitor (OG-L002) attenuated LPS tolerance when added during LPS stimulation, but not at the priming step (Fig. 2b). The expression profiles of LSD1 during LPS priming and LPS stimulation revealed increased protein expression during LPS priming while its level declined after resting period and LPS stimulation (Fig. 6a,b and Supplementary Fig. 8). The effects of LSD1 inhibition by OG-L002 on TNFα and IL-6 production during LPS tolerance were confirmed to be a dose dependent manner up to 40 μM (Fig. 6c,d). To understand whether LSD1 inhibition influenced histone modification of H3K4me3 at the promoters of these two genes, we performed a ChIP-qPCR. As shown in Fig. 6e,f, to our surprise, OG-L002 treatment did not significantly alter the level of H3K4me3 at the promoter of *tnf* but significantly reduced H3K4me3 association with the *Il6* promoter. This result suggests that LSD1 may mediate demethylation of other histone marks such as H3K9me3 during LPS tolerance that results in attenuated LPS tolerance.

**Figure 7:**
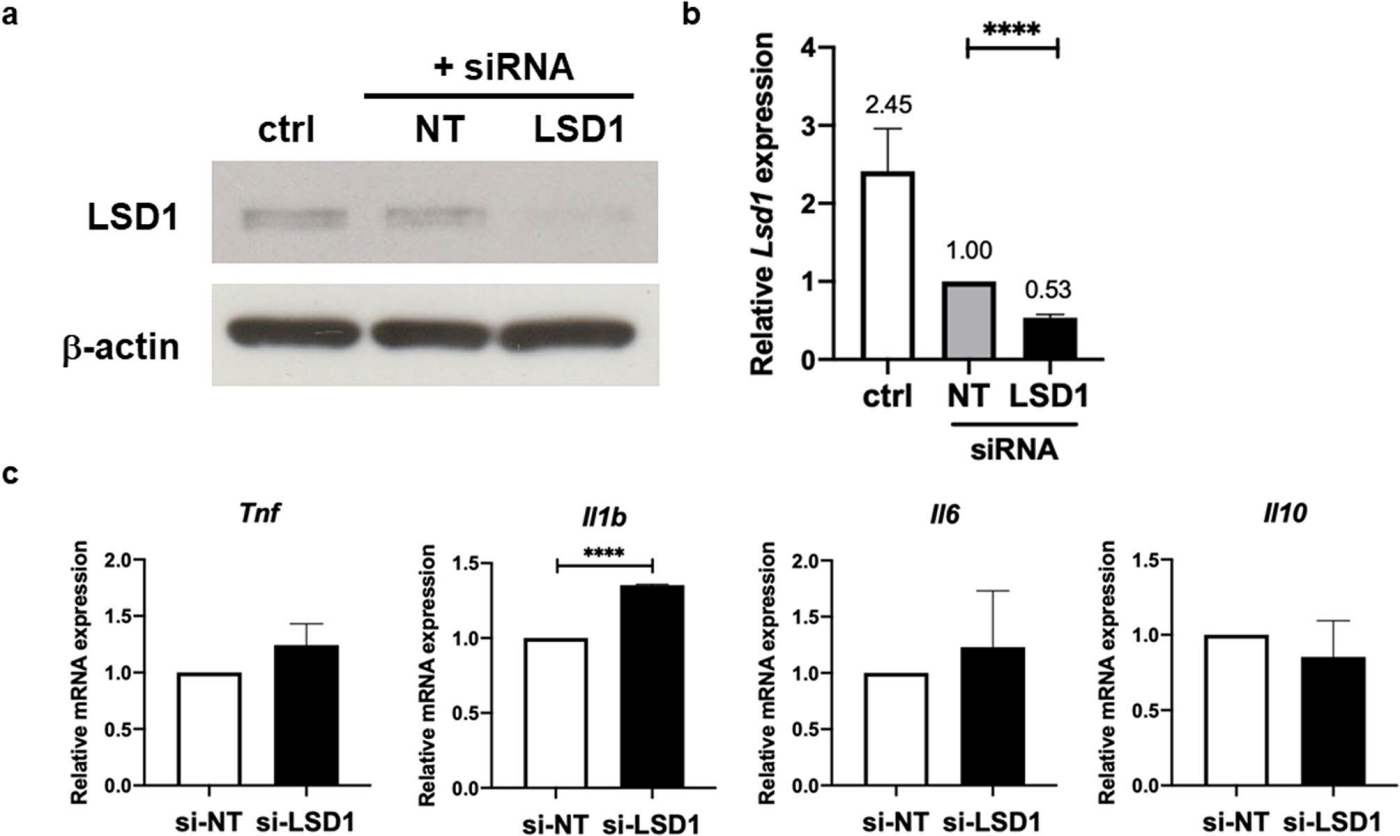
Effect of LSD1 silencing on LPS-tolerant macrophages. BMMs were transfected with 50 nM of siRNA *lsd1* or Non-Target (NT) siRNA as described in “Materials and methods”. (**a**, **b**) Level of LSD1 at 48 h after siRNA transfection was detected by Western blot and qRT-PCR. (**c)** Expression of pro-inflammatory and anti-inflammatory cytokines in *lsd1* silencing LPS-tolerant macrophages at 6 h after LPS stimulation. *, **, *** and **** indicate significant differences compared by two-tailed unpaired t-test at *p* < 0.05, *p* < 0.01, *p* < 0.001 and *p* < 0.0001, respectively.

Finally, we performed siRNA-mediated gene silencing of *lsd1* to confirm the results obtained by the use of inhibitor. As shown in Fig. 7a,b and Supplementary Fig. 8, siRNA targeting LSD1 effectively reduced LSD1 protein to roughly 50%. This siRNA treatment was applied to the LPS tolerance regimen described in the Supplementary Fig. 4. siRNA was transfected to BMM 48 h before LPS priming, followed by resting for 48 h and stimulation by LPS for 6 h. As shown in Fig. 7c, the level of *Il1b* mRNA significantly increased when LSD1 was silenced in comparison to the control non-targeting siRNA, an indicator that LPS tolerance is suppressed. Increased mRNA level of inflammatory genes, *tnf* and *Il6* but the difference did not reach statistical significance. Thus, inhibiting LSD1 by inhibitor or reducing its expression by siRNA treatment consistently rescued LPS tolerance by increasing tolerizable gene expression.

## Discussion

Trained immunity and tolerance in monocytes and macrophages are part of innate immune memory^10^. These innate immune memory phenomena are governed by transcription factors, epigenetic changes and metabolic rewiring that result in enhanced or suppressed responses in subsequent encounters with stimuli^17^. Because epigenetic reprogramming plays important roles in regulating innate immune memory, in this study, we aimed to identify novel epigenetic regulators that play a role in either trained or tolerance responses, which may potentially be a novel target for the treatment of conditions caused by dysregulated innate immune memory.

BG stimulation through Dectin1, Akt/mTOR and HIF-1α induced metabolic changes coupled with epigenetic reprogramming. This complicated regulatory network results in higher transcription of trained genes, including *Tnf*, *Il1b* and *Il6* (Supplementary Fig. 1a–e)^22,24^. In addition to BG, other microbial stimuli, such as BCG vaccination, and nonmicrobial stimuli, such as oxidized LDL, can also induce trained immunity in monocytes and macrophages^10^. Different trained stimuli utilize common mechanisms with some distinctive features for inducing trained immune responses. In this study, we used TNFα as a readout for the BG-trained response in macrophages because TNFα is one of the best characterized representative markers that is under the control of the trained immune response. Interestingly, only three compounds were identified in our screening assay that have an effect on the BG-trained immune response. An inhibitor of the histone methyltransferase SETD7, PFI-2 HCl, has a suppressive effect on trained immunity when added during BG priming but not during stimulation. A lysine methyltransferase, SETD7, has multiple histone and non-histone substrates that have been explored for targeted treatments of conditions, such as cancer and obesity^23^. Methylation of H3K4 mediated by SETD7 is associated with increased gene expression. A recent report identified SETD7 as a key enzyme that increases oxidative phosphorylation in BG-trained macrophages by upregulating key enzymes in the TCA cycle^18^. This result supports the validity of an unbiased screening approach in our study.

We also validated the impact of LSD1 inhibition on LPS tolerance by pharmacological and genetic approaches which yielded consistent outcomes. Both approaches showed that LSD1 play a positive role in regulating LPS tolerance. LSD1 is the key enzyme that mediates demethylation of mono- and di-methylated lysine, specifically H3K4 and H3K9 among others^25^. LSD1 functions downstream of LPS/TLR4 and controls acute inflammatory response during sepsis in myeloid cells. Deletion of LSD1 resulted in severe cytokine storm and lethality in sepsis^26^. In this study, we found that in LPS tolerance, inhibition of LSD1 during LPS stimulation but not LPS priming rescued LPS tolerance phenotype. However, we could not find changes in H3K4me3 level associated with the promoter of *Tnf* upon LSD1 inhibition whereas H3K4me3 level reduced in the *Il6* promoter. This result may indicate that LSD1 may mediate demethylation of other histone marks that have a combined effect on LPS tolerance. Other LSD1 substrate(s) that may play a crucial role in regulating LPS tolerance include H3K9, H3K27, H3K36, and H3K79^25^.

The other two compounds showed enhancing effects on BG-trained immunity, though lomeguatrib and carboplatin have not been previously associated with trained immunity. Lomeguatrib is a specific inhibitor of O^6^-methylguanine-DNA methyltransferase (MGMT). MGMT is a DNA repair protein that functions during DNA damage by alkylating agents and plays a role in conferring resistance to cancer cells against some cancer chemotherapies^27^. *Mgmt* knockout mice are susceptible to the lethal effect of alkylating agents^28^. In addition to its role in cancer, MGMT has been linked to inflammation, as hypermethylation of its promoter is associated with chronic inflammatory diseases and chronic infectious diseases^29–32^. How MGMT functions in epigenetic regulation and trained immunity requires further investigation.

LPS tolerance is accompanied by gene-specific chromatin modification that results in either suppression of gene transcription (including inflammatory genes) of tolerized genes and gene activation (including antimicrobial effector genes, Supplementary Figs. 1 and 2) or non-tolerized genes^5^. Upstream signaling molecules such as phosphatase SHIP-1 play important roles in reducing the phosphorylation of signal transduction molecules downstream of TLR^33^. In tolerized genes, histone deacetylation and certain lysine methylation cooperate to induce the state of transcriptional silencing^34^. In our screening assay, various inhibitors showed inhibitory effects against LPS tolerance, i.e., increasing TNFα production after repeated LPS stimulation. Histone-modifying enzyme inhibitors are a major group of inhibitors that reverse LPS tolerance. Several suppressive targets of these compounds have been characterized in previous studies, such as HDAC1 and HDAC3^35^, HDAC6^36^, and G9a and GLP^15,16^. Most of these inhibitors showed their effects when added during LPS stimulation after LPS priming. This result suggests that these molecules may function to rapidly modify epigenetic states that influence responses to LPS stimulation during the tolerance phase. Interestingly, inhibitors of histone demethylase, OG-L002, JIB-04 and ML-324, only showed an effect when added during LPS stimulation, but not during the LPS priming. This result indicated that histone demethylase activity may be essential for maintaining methylated histones to suppress chromatin for TNFα expression during LPS stimulation but not during LPS priming. The results from our screening assay showed that inhibitor function during LPS stimulation may open a window for reversing LPS tolerance after the first tolerogenic exposure and may be useful for rescuing the immune paralysis observed in conditions such as sepsis^37^.

In addition, the EZH2 histone methyltransferase inhibitor EPZ011989 enhanced TNFα production when added during priming or stimulation. EZH2 is a catalytic subunit of a large protein complex of PRC2 that regulates the methylation of the repressive histone mark H3K27me3. All EZH2 inhibitors in the current library (EPZ015666, GSK503, CPI-169, El1 and 3-DZNeP) function as S-adenosyl methionine competitive inhibitor. All of them contain pyridone-benzamide as a core structure and this core stucture is a target for cellular metabolism^38^. Although GSK503 and CPI-169 slightly rescued the TNFα production in LPS tolerance, EPZ015666 showed more than 1.5-fold changes of TNFα concentration over that of the vehicle control. Among these compounds, EPZ015666 is the only EZH2 inhibitor in our library that was designed to prevent oxidation by metabolism and possibly showed high potency in our assay. Ezh2 also methylates non-histone protein substrates such as suppressor of cytokine signaling 3 (SOCS3) and the transcription factor GATA4^39,40^. The wide ranges of the substrates of PRC2/EZH2 imply that PRC2/EZH2 may regulate multiple steps during LPS tolerance.

To our surprise, several DNA/RNA synthesis inhibitors showed inhibitory effects against LPS tolerance and enhancing effects against the BG-trained immune response. In addition, Aurora kinase inhibitors were among the compounds that reversed LPS tolerance (Fig. 2b). When protein interaction analysis was performed, apoptosis- and DNA repair-related genes, such as Trp53 and Msh6, were indicated in the trained immunity network (Fig. 3b). Protein interaction analysis revealed two specific roles of Aurora kinases in the regulation of cell division and epigenetic regulation (Fig. 3b). Both DNA/RNA synthesis inhibitors and Aurora kinase inhibitors may not directly link to epigenetic processes but there are evidences supporting that DNA damage may alter epigenetic states in trained immunity^41^ while Aurora kinases also classify as enzymes that phosphorylate serine residue in histone such as H3S10 and H3S28^42,43^. Therefore, the findings here may uncover novel targets that modify epigenetics during innate immune memory.

Aurora kinases are well characterized in regulating mitotic processes, and their inhibition results in cytokinesis failure and is one of the targets for cancer therapy^44^. Because priming with LPS did not induce cell proliferation, Aurora kinases may regulate LPS tolerance through other mechanisms not related to cell cycle regulation, such as epigenetic regulation (Supplementary Fig. 5). Among the three subtypes of Aurora kinases, Aurora kinase B has been reported to regulate the deposition of some repressive histone marks, such as phosphorylation of H3S10, H3S28 and H3K9me3^42,45^. Interestingly, Aurora kinase B was the only subtype that significantly changed its phosphorylation level during LPS tolerance. Increased phosphorylation of Aurora kinase B during the resting period of LPS tolerance may regulate the deposition of these repressive histone marks. In addition to its roles during cell division and epigenetic regulation, Aurora kinase A participates in early signaling during T cell activation by regulating CD3ζ-containing vesicle trafficking^46^. In one report, Aurora kinase A regulated M1 macrophage polarization by suppressing NF-κB activation and switched macrophages toward the M2 phenotype^47^. How these groups of enzymes regulate LPS tolerance needs further investigation. DNA/RNA synthesis inhibitors included in this screening are often used as chemotherapeutics against cancers, such as the platinum derivative carboplatin. They induce cancer cell death by various mechanisms^48^. However, the cell cycle and cell death have not been investigated in terms of training or tolerance in macrophages, but it is possible that innate immune memory is tightly coupled with epigenetic modification and cell cycle/cell death.

Using STRING database, we uncovered clusters of protein networks of the inhibitor targets identified in this study. For LPS tolerance response, histone modification and chromatin modifying enzymes formed a large cluster with targets identified in this study such as LSD1, PRMT3, PRMT5, EZH2, JMJD2 and SIRT1. Most of these interactions are experimentally determined. Proteins involved in base-excision repair (PARP1/2) and cell division (Aurka A/B/C) formed small clusters that linked to the histone modification cluster via EZH2, SIRT1 and HDAC6. For the BG-trained response, two clusters of proteins in chromatin organization and cellular response to DNA damage were linked together via TRP53 and SETD7. Although TRP53 was not identified in our screening, methylation of TRP53 by SETD7 (Set7/9) has been reported in cancer settings^49^. Thus, BG-trained immunity may involve modification by methyltransferase of non-histone substrates^50^. Some of the links shown here are based on the curated database and require further experimental prove for the physical/functional interactions in innate immune memory.

Tolerance and trained innate immune memory are tightly regulated, and the interaction between the two events has been reported at multiple levels. BG treatment is able to revert the epigenetic states conditioned by LPS tolerance, and trained immunity may be a mechanistic link between sepsis and atherosclerosis^13,51^. Recent emerging evidence has pointed to the critical roles of innate immune memory in various pathological conditions, including chronic inflammatory diseases and cancer. The use of epigenetic modifying compounds provides potential interventions for such diseases. The limitation of this study is that the observed effect of each compound on innate immune memory may be the result of side-effect of the compound and this point needs to be further validated by genetic approaches. Furthermore, our screening results provide new unappreciated key enzymes/pathways that may regulate training and tolerance in macrophages.

## Supplementary Information

Supplementary Information.
