# Supplementary material for: Screening of compounds to identify novel epigenetic regulatory factors that affect innate immune memory in macrophages
Source: Sci Rep. 2022 Feb 3;12:1912. doi: 10.1038/s41598-022-05929-x (PMC8814160; doi:10.1038/s41598-022-05929-x)
Supplement: Supplementary file 1 — Supplementary Information. [file 41598_2022_5929_MOESM1_ESM.docx]

**Supplementary Information**

**Screening of Compounds to Identify Novel Epigenetic Regulatory Factors in Innate Immune Memory in Macrophages**

**Salisa Benjaskulluecha^1, 2^, Atsadang Boonmee ^2, 3^, Thitiporn Pattarakankul ^3, 4^, Benjawan Wongprom ^2, 3^, Jeerameth Klomsing ^3^, and Tanapat Palaga* ^1, 2, 3^**

^1^ Interdisciplinary Graduate Program in Medical Microbiology, Graduate School, Chulalongkorn University, Bangkok, 10330, Thailand

^2^ Center of Excellence in Immunology and Immune-Mediated Diseases, Chulalongkorn University, Bangkok, 10330, Thailand

^3^ Department of Microbiology, Faculty of Science, Chulalongkorn University, Bangkok, 10330, Thailand

^4^ Center of Excellence in Advanced Materials and Biointerfaces, Chulalongkorn University, Bangkok, 10330, Thailand

**^*^**tanapat.p@chula.ac.th


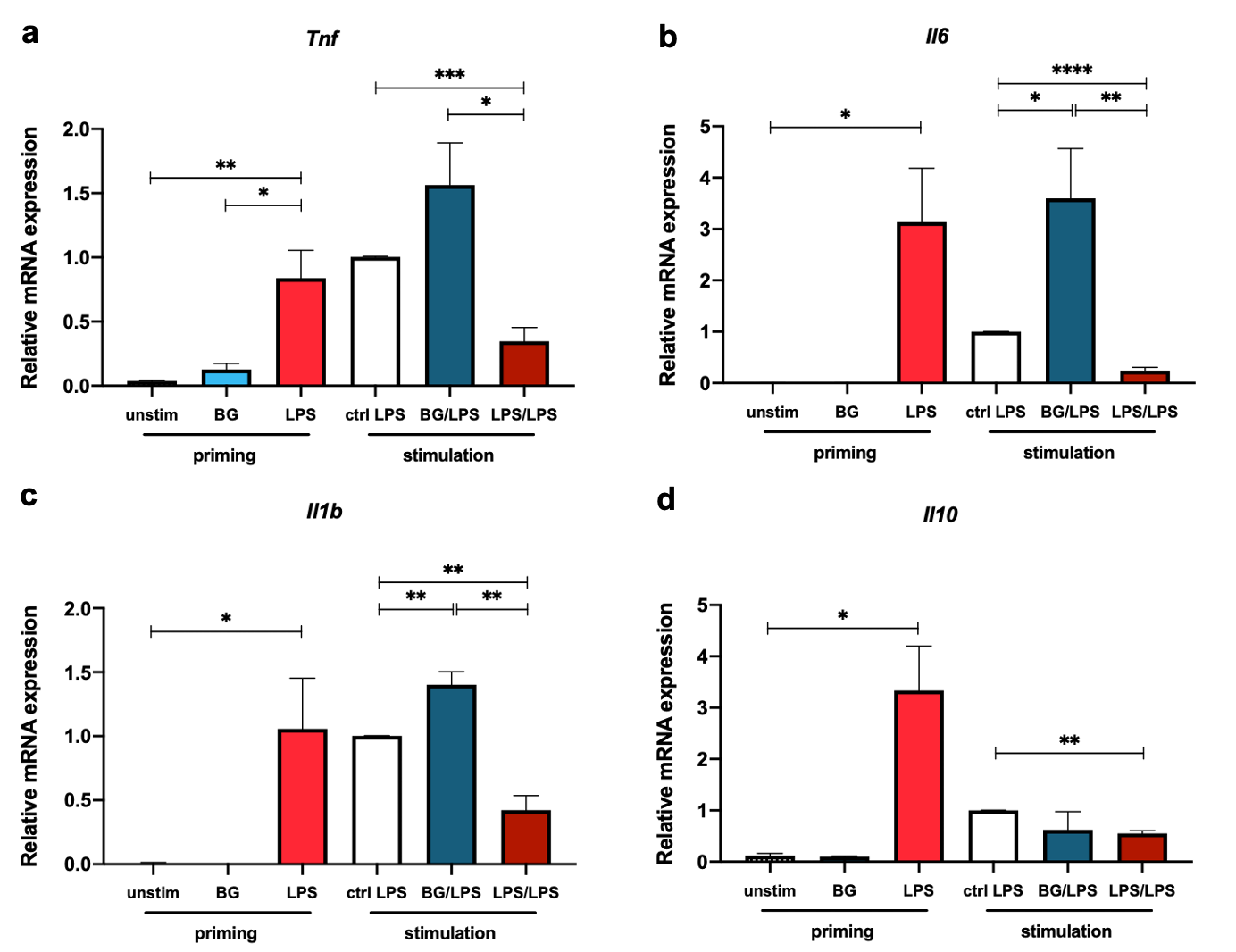
 **Supplementary Figure 1** Expression profiles of pro-inflammatory and anti-inflammatory cytokines in BG-trained and LPS-tolerant macrophages

BG-trained and LPS-tolerant macrophages were prepared as described in Figure 1a. (**a-d**) mRNA expression of proinflammatory cytokines *Tnf*, *Il6* and *Il1b* and anti-inflammatory cytokine *Il10* were detected after the priming and stimulation for 6 hr. The relative expression levels of cytokine genes was normalized to the expression of *Actb* by the 2^-∆∆CT^ method and calculated by comparing with the BMMs with LPS stimulation (ctrl LPS). *, **, and *** indicates statistically significant difference by two-tailed unpaired t-test at *p*< 0.05, *p*<0.01 and *p*<0.001, respectively.

**Supplementary Figure 2** Characterization of BG-trained and LPS-tolerant macrophages

BG-trained and LPS-tolerant macrophages were prepared as described in Figure 1a. (**a-b**) The phosphorylated and total mTOR protein were detected by Western blot as indicated in Figure 1c. The relative intensity of phosphorylated mTOR from Western blot was quantitated by ImageJ analysis and normalized to total mTOR. (**c-f**) Expression profiles of tolerizeable genes (T-genes) and non-tolerizeable genes (NT-genes) were detected in LPS-tolerant macrophages after stimulation for 6 hr with LPS. The relative mRNA expression was normalized to the expression of *Actb* by the 2^-∆∆CT^ method and calculated by comparing with LPS-stimulated BMM (-/LPS). *, **, *** and **** indicate statistical significant difference by one-way ANOVA with Tukey’s multiple comparison test at *p*< 0.05, *p*<0.01, *p*<0.001 and *p*<0.0001, respectively.


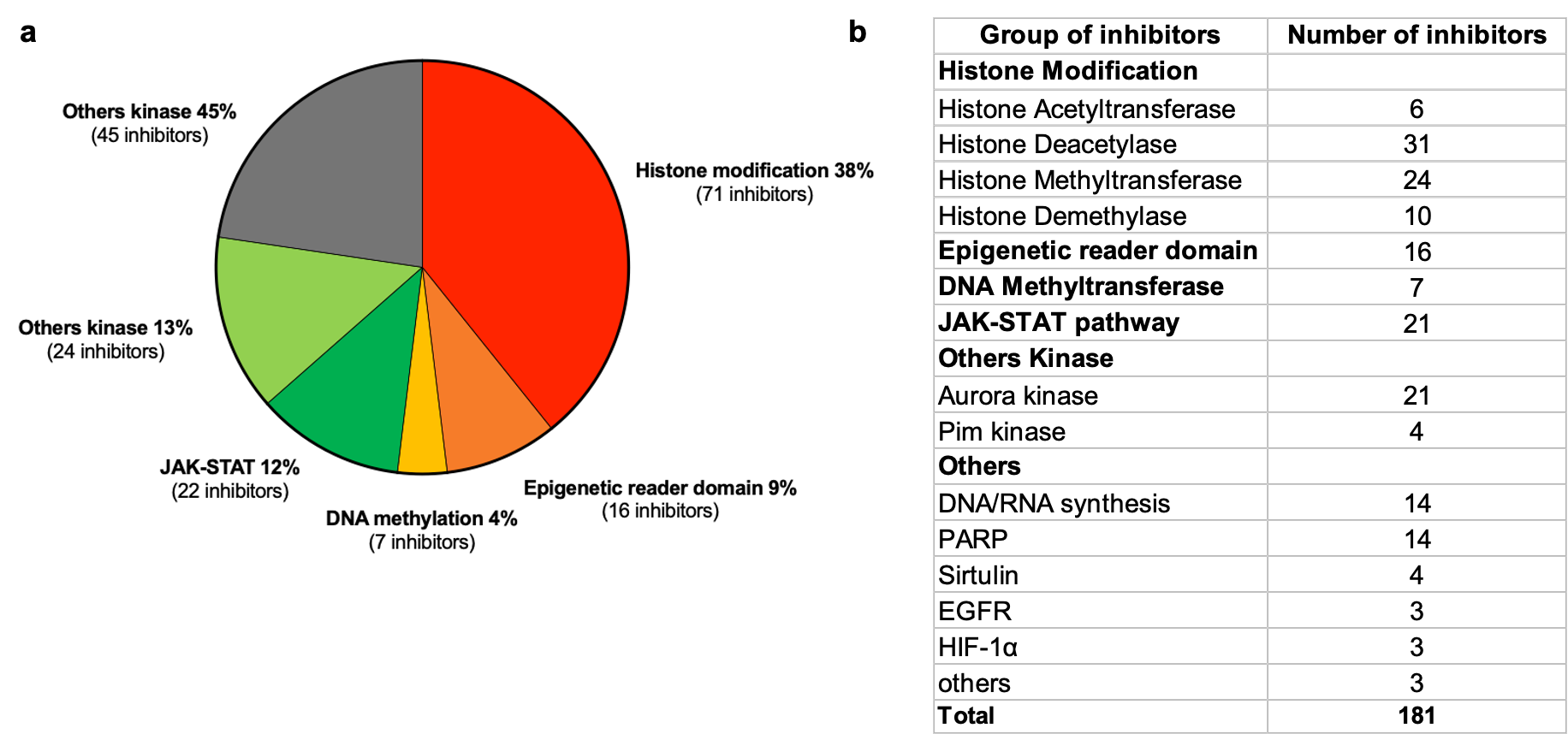


**Supplementary Figure 3.** Composition of the epigenetics compound library

**a.** Pie chart showed the distribution of targets of the compounds in the library used in this study and detailed targets of the compounds. **b.** Numbers of compounds in each category.


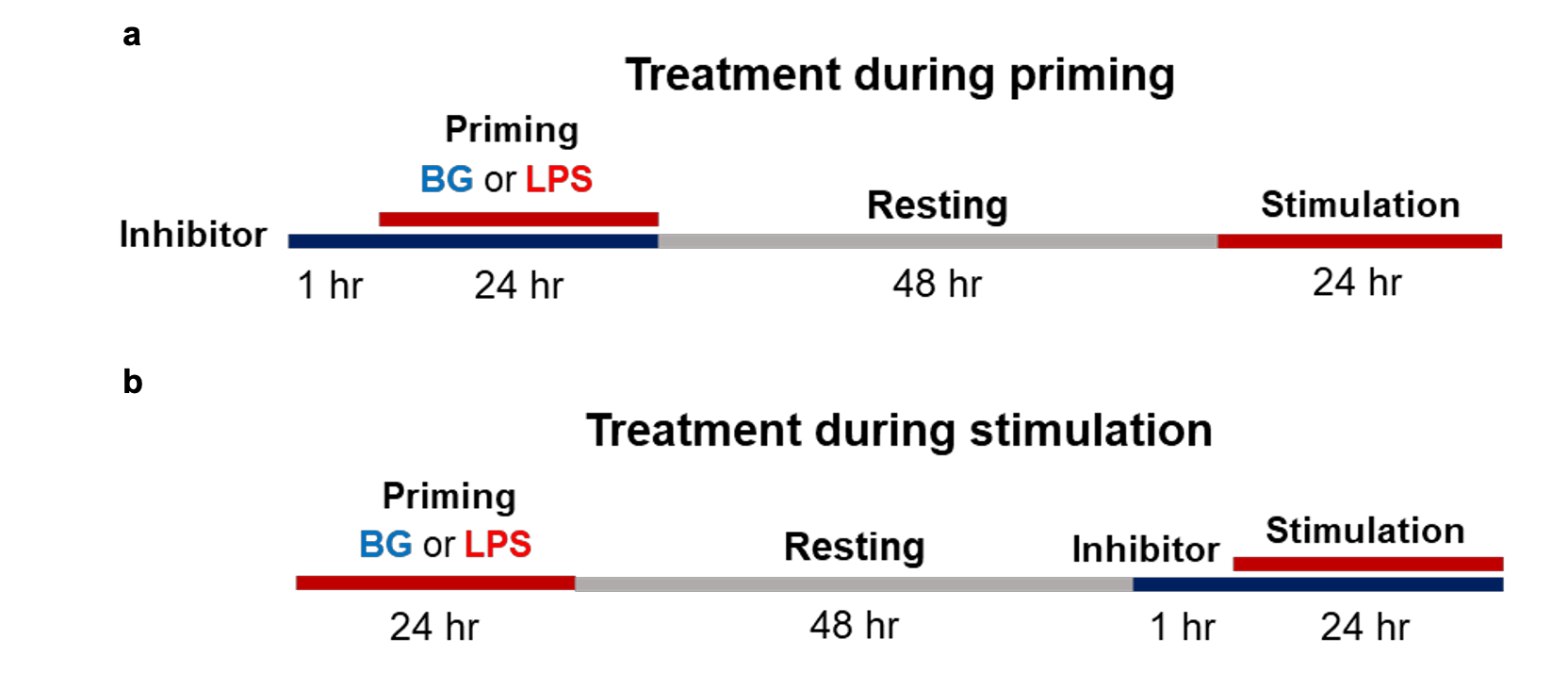


**Supplementary Figure 4.** The screening protocols to identify compounds with enhancing or suppressing TNFα production in BG-trained or LPS-tolerant macrophages

The assay was performed in two ways. For the pre-treatment during priming strategy, the compounds were added during the priming step (**a**) while for the pre-treatment during the stimulation strategy, the compounds were added during stimulation step after BG or LPS priming and resting (**b**).


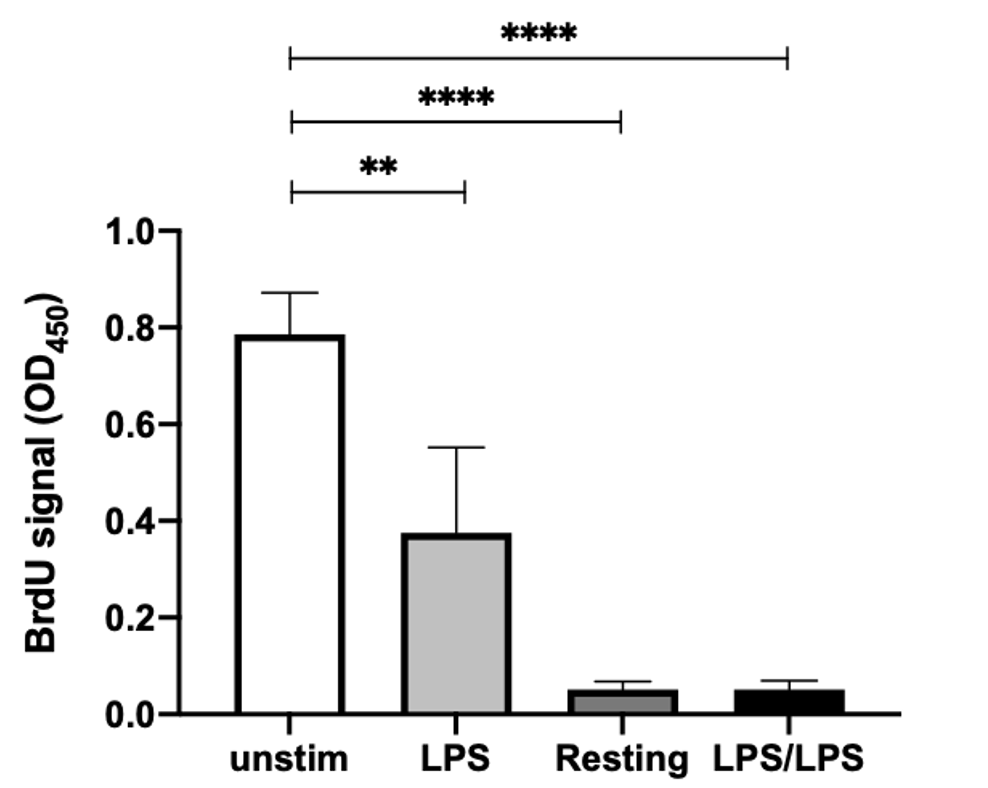


**Supplementary Figure 5.** Cell proliferation in LPS-tolerant macrophages.

BMMs were treated as indicated for LPS-tolerant macrophages. Proliferation was measured by BrdU uptake. *, **, *** and **** indicate statistically significant difference by one-way ANOVA with Dunnett’s multiple comparison test at *p*< 0.05, *p*<0.01, *p*<0.001 and *p*<0.0001, respectively.

**Supplementary Figure 6.** Representative Western blot of H3K4me3, H3K27me3 and total H3 from Figure 1e and 1g

**Supplementary Figure 7.** Representative Western blot of phosphor-Aurora kinase A/B/C, SETD7 and β-actin from Figure 4d and 5c

**Supplementary Figure 8.** Representative Western blot of LSD1 and β-actin from Figure 6a and 7a


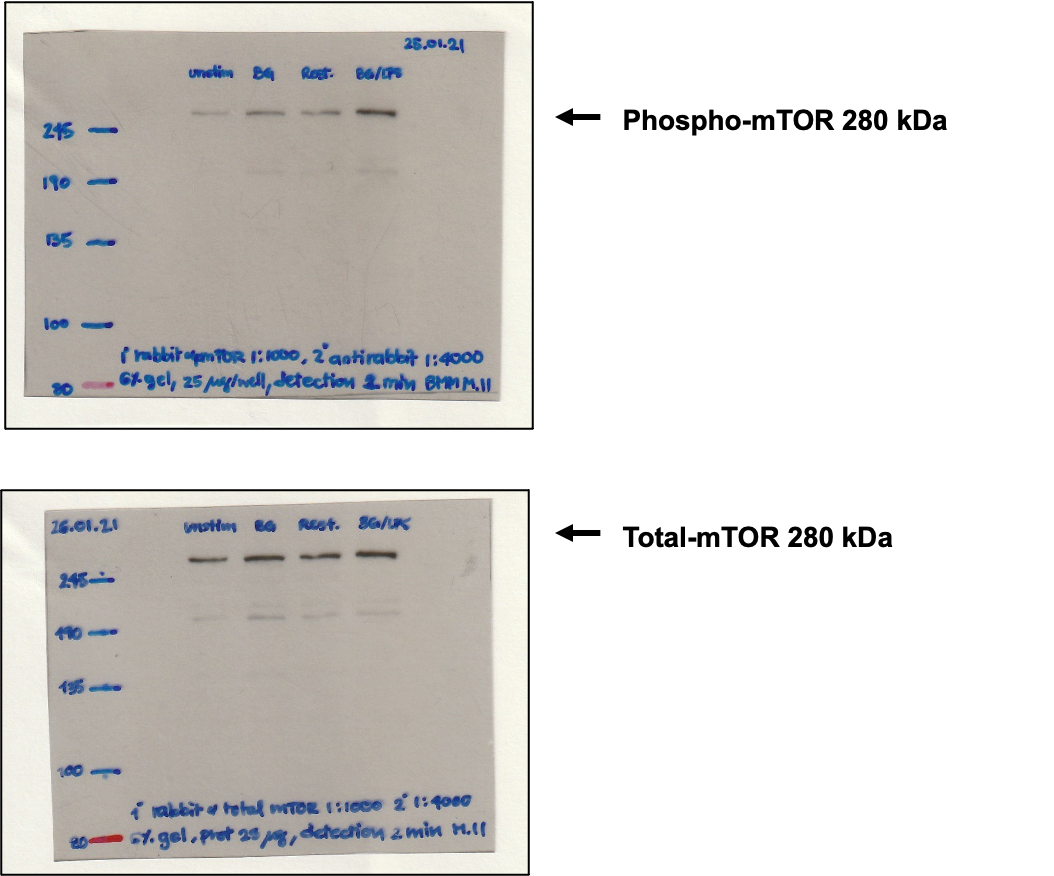


**Supplementary Figure 9.** Representative Western blot of phospho and total mTOR from Supplementary Figure 2a

**Supplementary Table 1.** List of Compounds in the Epigenetics Compounds Library used in this study (Selleckchem).

| **Catalog Number** | **Product Name** |
| --- | --- |
| S1004 | Veliparib (ABT-888) |
| S1007 | Roxadustat (FG-4592) |
| S1030 | Panobinostat (LBH589) |
| S1045 | Trichostatin A (TSA) |
| S1047 | Vorinostat (SAHA, MK0683) |
| S1048 | Tozasertib (VX-680, MK-0457) |
| S1053 | Entinostat (MS-275) |
| S1060 | Olaparib (AZD2281, Ku-0059436) |
| S1085 | Belinostat (PXD101) |
| S1087 | Iniparib (BSI-201) |
| S1090 | Abexinostat (PCI-24781) |
| S1095 | Dacinostat (LAQ824) |
| S1096 | Quisinostat (JNJ-26481585) 2HCl |
| S1098 | Rucaparib (AG-014699,PF-01367338) phosphate |
| S1100 | MLN8054 |
| S1103 | ZM 447439 |
| S1107 | Danusertib (PHA-739358) |
| S1122 | Mocetinostat (MGCD0103) |
| S1129 | SRT1720 HCl |
| S1132 | INO-1001 (3-Aminobenzamide) |
| S1133 | Alisertib (MLN8237) |
| S1134 | AT9283 |
| S1143 | AG-490 (Tyrphostin B42) |
| S1147 | Barasertib (AZD1152-HQPA) |
| S1154 | SNS-314 Mesylate |
| S1168 | Valproic acid sodium salt (Sodium valproate) |
| S1171 | CYC116 |
| S1181 | ENMD-2076 |
| S1194 | CUDC-101 |
| S1200 | Decitabine |
| S1216 | PFI-1 (PF-6405761) |
| S1233 | 2-Methoxyestradiol (2-MeOE2) |
| S1249 | JNJ-7706621 |
| S1327 | Ellagic acid |
| S1378 | Ruxolitinib (INCB018424) |
| S1393 | Pirarubicin |
| S1396 | Resveratrol |
| S1422 | Droxinostat |
| S1451 | Aurora A Inhibitor I |
| S1454 | PHA-680632 |
| S1463 | Ofloxacin |
| S1484 | MC1568 |
| S1509 | Norfloxacin |
| S1515 | Pracinostat (SB939) |
| S1529 | Hesperadin |
| S1541 | Selisistat (EX 527) |
| S1782 | Azacitidine |
| S2012 | PCI-34051 |
| S2018 | ENMD-2076 L-(+)-Tartaric acid |
| S2158 | KW-2449 |
| S2162 | AZD1480 |
| S2170 | Givinostat (ITF2357) |
| S2178 | AG-14361 |
| S2179 | Gandotinib (LY2784544) |
| S2198 | SGI-1776 free base |
| S2214 | AZ 960 |
| S2219 | Momelotinib (CYT387) |
| S2244 | AR-42 |
| S2391 | Quercetin |
| S2554 | Daphnetin |
| S2627 | Tubastatin A HCl |
| S2686 | NVP-BSK805 2HCl |
| S2692 | TG101209 |
| S2693 | Resminostat |
| S2718 | TAK-901 |
| S2719 | AMG-900 |
| S2736 | Fedratinib (SAR302503, TG101348) |
| S2740 | GSK1070916 |
| S2759 | CUDC-907 |
| S2770 | MK-5108 (VX-689) |
| S2779 | M344 |
| S2789 | Tofacitinib (CP-690550,Tasocitinib) |
| S2796 | WP1066 |
| S2804 | Sirtinol |
| S2806 | CEP-33779 |
| S2818 | Tacedinaline (CI994) |
| S2821 | RG108 |
| S2851 | Baricitinib (LY3009104, INCB028050) |
| S2867 | WHI-P154 |
| S2886 | PJ34 |
| S2902 | S-Ruxolitinib (INCB018424) |
| S2919 | IOX2 |
| S3001 | Clevudine |
| S3147 | Entacapone |
| S4125 | Sodium Phenylbutyrate |
| S4246 | Tranylcypromine (2-PCPA) HCl |
| S4294 | Procainamide HCl |
| S5001 | Tofacitinib (CP-690550) Citrate |
| S7029 | AZD2461 |
| S7036 | XL019 |
| S7041 | CX-6258 HCl |
| S7062 | Pinometostat (EPZ5676) |
| S7070 | GSK J4 HCl |
| S7079 | SGC 0946 |
| S7088 | UNC1215 |
| S7104 | AZD1208 |
| S7110 | (+)-JQ1 |
| S7113 | Zebularine |
| S7120 | 3-deazaneplanocin A (DZNeP) HCl |
| S7152 | C646 |
| S7189 | I-BET-762 |
| S7229 | RGFP966 |
| S7231 | GSK2801 |
| S7233 | Bromosporine |
| S7234 | IOX1 |
| S7237 | OG-L002 |
| S7238 | NVP-TNKS656 |
| S7256 | SGC-CBP30 |
| S7265 | MM-102 |
| S7276 | SGI-1027 |
| S7281 | JIB-04 |
| S7292 | RG2833 (RGFP109) |
| S7294 | PFI-2 HCl |
| S7295 | RVX-208 |
| S7296 | ML324 |
| S7300 | PJ34 HCl |
| S7304 | CPI-203 |
| S7305 | MS436 |
| S7315 | PFI-3 |
| S7324 | TMP269 |
| S7353 | EPZ004777 |
| S7360 | OTX015 |
| S7373 | UNC669 |
| S7438 | ME0328 |
| S7473 | Nexturastat A |
| S7476 | MG149 |
| S7541 | Decernotinib (VX-509) |
| S7555 | 4SC-202 |
| S7570 | UNC0379 |
| S7572 | A-366 |
| S7574 | GSK-LSD1 2HCl |
| S7581 | GSK J1 |
| S7582 | Anacardic Acid |
| S7591 | BRD4770 |
| S7605 | Filgotinib (GLPG0634) |
| S7610 | UNC0631 |
| S7611 | EI1 |
| S7616 | CPI-169 |
| S7618 | MI-2 (Menin-MLL Inhibitor) |
| S7619 | MI-3 (Menin-MLL Inhibitor) |
| S7620 | GSK1324726A (I-BET726) |
| S7625 | Niraparib (MK-4827) tosylate |
| S7641 | Remodelin |
| S7656 | CPI-360 |
| S7680 | SP2509 |
| S7681 | OF-1 |
| S7748 | EPZ015666(GSK3235025) |
| S7767 | AZ6102 |
| S7795 | ORY-1001 (RG-6016) 2HCl |
| S7796 | GSK2879552 2HCl |
| S7804 | GSK503 |
| S7805 | EPZ011989 |
| S7832 | SGC707 |
| S7835 | I-BRD9 |
| S8001 | Ricolinostat (ACY-1215) |
| S8004 | ZM 39923 HCl |
| S8005 | SMI-4a |
| S8006 | BIX 01294 |
| S8038 | UPF 1069 |
| S8043 | Scriptaid |
| S8049 | Tubastatin A |
| S8056 | Lomeguatrib |
| S8057 | Pacritinib (SB1518) |
| S8096 | Mirin |
| S8111 | GSK591 |
| S8112 | MS023 |
| S8146 | Mitomycin C |
| S8179 | BI-7273 |
| S8180 | PF-CBP1 HCl |
| S8195 | Oclacitinib |
| S8209 | HLCL-61 HCL |
| S8323 | ITSA-1 (ITSA1) |
| S1149 | Gemcitabine HCl |
| S1215 | Carboplatin |
| S1373 | Daptomycin |
| S1384 | Mizoribine |
| S1648 | Cytarabine |
| S1826 | Nedaplatin |
| S1995 | Procarbazine HCl |
| S7419 | Blasticidin S HCl |
| S8197 | APTSTAT3-9R |

**Supplementary Table 2.** List of primers used in this study.

| Gene | Forward (F) and Reverse (R) Primers | Annealing Temperature | Reference |
| --- | --- | --- | --- |
| *Tnf* | F: 5’-AGC CCA CGT CGT AGC AAA CCA C-3’  R: 5’-ATC GGC TGG CAC CAC TAG TTG GT-3’ | 55 °C | Viceconte *et al*.^1^ |
| *Il6* | F: 5’-CTC TGG GAA ATC GTG GAA ATG-3’  R: 5’-AAG TGC ATC ATC GTT GTT CAT ACA-3’ | 57 °C | Minashima *et al*.^2^ |
| *Il1b* | F: 5’-TAT ACC TGT CCT GTG TAA-3  R: 5’-TTG ACT TCT ATC TTG TTG A-3’ | 53 °C | Lai *et al*. ^3^ |
| *Il10* | F: 5’-TCA AAC AAA GGA CCA GCT GGA CAA CAT ACT GC-3’  R: 5’-CTG TCT AGG TCC TGG AGT CCA GCA GAC TCA A-3’ | 58 °C | Palaga *et al*.^4^ |
| *Cd40* | F: 5’-GTT TAA AGT CCC GGA TGC GA-3’  R: 5’-CTC AAG GCT ATG CTG TCT GT-3’ | 64 °C | Magner *et al*.^5^ |
| *Serpine1* | F: 5’-TCA TCA ATG ACT GGG TGG AA-3’  R: 5’-TGC TGG CCT CTA AGA AAG GA-3’ | 62 °C | Syed *et al*.^6^ |
| *Marco* | F: 5’-GAA GAC TTC TTG GGC AGC AC-3’  R: 5’-CTT CTT GGG CAC TGG ATC AT-3’ | 62 °C | Jing *et al*.^7^ |
| *Camp* | F: 5’-CGA GCT GTG GAT GAC TTC AA-3’  R: 5’-CAG GCT CGT TAC AGC TGA TG-3’ | 68 °C | Kin *et al*.^8^ |
| *Setd7* | F: 5’-CAG CCG CCA TGG ATA GCG ACG-3’  R: 5’-CTC CAG GGT GCT GCC GTC AAA G-3’ | 62 °C | Kofent *et al*.^9^ |
| *Actb* | F: 5’-ACC AAC TGG GAC GAC ATG GAG AA-3’  R: 5’-GTG GTG GTG AAG CTG TAG CC-3’ | 55 °C | Wongchana *et al*.^10^ |
| *mTnf* promoter | F: 5’-CAA CTT TCC AAA CCC TCT GC-3’  R: 5’-CTG GCT AGT CCC TTG CTG TC-3’ | 59 °C | Saz-Leal *et al*.^11^ |
| *mIl6*  promoter | F: 5’-CAC TTC ACA AGT CGG AGG CT-3’  R: 5’-AAT GAA TGG ACG CCC ACA CT-3’ | 61 °C | Hu *et al*.^12^ |

**Supplementary Table 3.**  Dose of effective inhibitors and concentration of TNFα from BG-trained macrophages from Figure 2a

**Supplementary Table 4.** Dose of effective inhibitors and concentration of TNFα from LPS-tolerant macrophages from Figure 2b

**References**

1 Viceconte, N. *et al.* Neuromelanin activates proinflammatory microglia through a caspase-8-dependent mechanism. *J Neuroinflammation* **12**, 5, doi:10.1186/s12974-014-0228-x (2015).

2 Minashima, T., Campbell, K. A., Hadley, S. R., Zhang, Y. & Kirsch, T. The role of ANK interactions with MYBBP1a and SPHK1 in catabolic events of articular chondrocytes. *Osteoarthritis Cartilage* **22**, 852-861, doi:10.1016/j.joca.2014.04.008 (2014).

3 Lai, C. F. *et al.* Blockade of cysteine-rich protein 61 attenuates renal inflammation and fibrosis after ischemic kidney injury. *Am J Physiol Renal Physiol* **307**, F581-592, doi:10.1152/ajprenal.00670.2013 (2014).

4 Palaga, T. *et al.* Notch signaling is activated by TLR stimulation and regulates macrophage functions. *European Journal of Immunology* **38**, 174-183, doi:10.1002/eji.200636999 (2008).

5 Magner, W. J. *et al.* Activation of MHC class I, II, and CD40 gene expression by histone deacetylase inhibitors. *J Immunol* **165**, 7017-7024, doi:10.4049/jimmunol.165.12.7017 (2000).

6 Syed, K. M. *et al.* Correction: Histone chaperone APLF regulates induction of pluripotency in murine fibroblasts (doi:10.1242/jcs.194035). *J Cell Sci* **131**, doi:10.1242/jcs.218842 (2018).

7 Jing, J. *et al.* Role of macrophage receptor with collagenous structure in innate immune tolerance. *J Immunol* **190**, 6360-6367, doi:10.4049/jimmunol.1202942 (2013).

8 Kin, N. W., Chen, Y., Stefanov, E. K., Gallo, R. L. & Kearney, J. F. Cathelin-related antimicrobial peptide differentially regulates T- and B-cell function. *Eur J Immunol* **41**, 3006-3016, doi:10.1002/eji.201141606 (2011).

9 Kofent, J., Zhang, J. & Spagnoli, F. M. The histone methyltransferase Setd7 promotes pancreatic progenitor identity. *Development* **143**, 3573-3581, doi:10.1242/dev.136226 (2016).

10 Wongchana, W., Lawlor, R. G., Osborne, B. A. & Palaga, T. Impact of Notch1 Deletion in Macrophages on Proinflammatory Cytokine Production and the Outcome of Experimental Autoimmune Encephalomyelitis. *J Immunol* **195**, 5337-5346, doi:10.4049/jimmunol.1401770 (2015).

11 Saz-Leal, P. *et al.* Targeting SHIP-1 in Myeloid Cells Enhances Trained Immunity and Boosts Response to Infection. *Cell Rep* **25**, 1118-1126, doi:10.1016/j.celrep.2018.09.092 (2018).

12 Hu, L. *et al.* Epigenetic Regulation of Interleukin 6 by Histone Acetylation in Macrophages and Its Role in Paraquat-Induced Pulmonary Fibrosis. *Front Immunol* **7**, 696, doi:10.3389/fimmu.2016.00696 (2016).
